# Intermittent Abdominal Pressure Ventilation: An Alternative for Respiratory Support

**DOI:** 10.1155/2021/5554765

**Published:** 2021-08-23

**Authors:** Giuseppe Fiorentino, Anna Annunziata, Antonietta Coppola, Antonella Marotta, Francesca Simioli, Pasquale Imitazione, Maurizia Lanza, Rosa Cauteruccio, Antonio M. Esquinas

**Affiliations:** ^1^Sub-intensive Care Unit, Department of Respiratory Pathophysiology and Rehabilitation Monaldi—A.O. Dei Colli, Monaldi Hospital, Naples, Italy; ^2^Hospital General Universitario Morales Meseguer, Murcia, Spain

## Abstract

Intermittent abdominal pressure ventilation is a positive pressure ventilation technique that works with abdominal compressions. It has been known since 1938; however, for many years, it was out of production. In recent years, a new device has been produced that has captured the attention to this old respiratory support technique. We considered eight patients with respiratory failure secondary to a neuromuscular disease (congenital myopathy, Duchenne dystrophy, and amyotrophic lateral sclerosis) intolerant to daytime noninvasive ventilation (NIV). IAPV was proposed as an alternative to NIV. We performed baseline and post-IAPV respiratory function assessment. All patients, two years later, are still using intermittent abdominal ventilation. Intermittent positive abdominal mechanical ventilation can be a valid alternative to noninvasive mechanical ventilation with a nasal or face mask. It improves gas exchange, symptoms, and quality of life, decreases the incidence of pneumonia, and can avert the need for intubation and tracheotomy.

## 1. Background

We are used to thinking of noninvasive mechanical ventilation as positive pressure ventilation using a nasal or face mask. This type of interface interferes with the patient's quality of life who has to start using NIV, and often, the patient rejects it. Intermittent abdominal pressure ventilation is a positive pressure ventilation technique that works with abdominal compressions. It is a system of noninvasive respiratory care known since 1938 [1], individually modified to the cure of postdiphtheritic respiratory paralysis or respiratory paralysis due to anterior poliomyelitis [2]. In 1987, a marginal approach to NIV with IAPV was described in patients with spinal cord injury [3].

In 1988, Miller et al. described a rehabilitative practice in high quadriplegic patients with tracheostomy about speech capability and safe respiratory management with an optimal patient tolerance of treatment [4]. Later, in 1991, Bach described long-term use of IAPV in patients diagnosed with different neuromuscular diseases (myopathy, Duchenne dystrophy, and spinal cord injury). In this paper, only 54 out of 209 initially undergoing the trial were long-term adapted to IAPV [2].

IAPV facilitates diaphragmatic motion and may be particularly useful in patients with bilateral diaphragmatic weakness or paralysis and permits plugging of the tracheostomy tube with cuff deflation for several hours during the day, with the prevention of tracheal damage. However, it was out of production for many years, until 1990. It led to a loss of knowledge of the method, and only in recent years, some centres have begun to have an interest in this old, renewed technology again. The device available today is the LunaBelt (Dima Italia Inc., Bologna, Italy); it is a transportable ventilator.

Along with the PBAir™ corset, it is easy to utilize the intermittent abdominal pressure ventilation (IAPV) device. The LunaBelt is a portable ventilator explicitly designed to operate the IAPV through dedicated software and a new abdominal interface called PBAir^®^. Recently, case reports have been published on its use in patients with late-onset Pompe disease, postischemic cervical myelopathy, and ALS [5–7]. IAPV has been reported to facilitate good mechanical ventilation adaptation with an efficient ventilation pattern and good peripheral oxygenation. We describe the use of IAPV in our respiratory pathophysiology unit.

## 2. Materials and Methods

**Figure 1:**
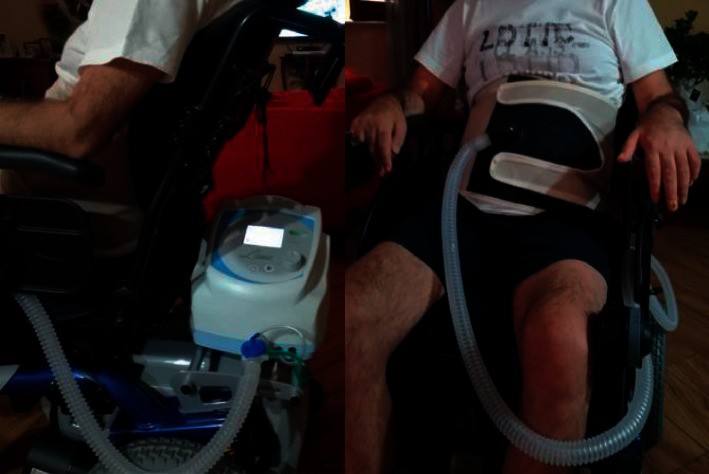
Patient during ventilation with LunaBelt.

We evaluated 8 patients (Pt) diagnosed with neuromuscular disease who presented with ventilatory insufficiency with dyspnea and reduced tidal volume on spirometry and with an indication for NIV. One congenital myopathy patient (Pt 1, female, 32 ys), two Duchenne muscular dystrophy patients (Pt 2, 3, males, 22 and 20 ys), and two ALS patients (Pt 4, 5, males, 62 and 63 ys) had previously refused noninvasive mechanical ventilation due to claustrophobia, interface intolerance, and emotional and psychological factors. Pt 2, affected by Duchenne muscular dystrophy, also complained of gastric and colonic distension. Two patients with ALS (Pt 6, 7, males, 68 and 25 ys) and one patient with Duchenne muscular dystrophy (Pt 8, male, 19 ys) were treated with noninvasive mechanical ventilation with a nasal mask, with poor compliance of gastric hyperdistention and severe skin decubitus (Table 1). All patients agreed to carry out a trial with IAPV with LunaBelt (Dima Italia Inc., Bologna, Italy). The LunaBelt has internal battery power that can also, eventually, be used for noninvasive respiratory support for sleep. It provides a dedicated IAPV mode. The IAPV corset is lightweight, comfortable, and fitted with Velcro fasteners (Figure 1). Like earlier IAPV, cyclical inflation of a rubber bladder inside the corset pushes the diaphragm upwards to eject air from the residual volume. It allows air to enter the lungs via the upper airway as gravity moved the diaphragm back to its resting position [8, 9]. We set the following IAPV parameters: pressure (pressure inside the bladder), inspiratory time (an adequate inspiratory time when the diaphragm returns), frequency (respiratory rate), and rise time (time to pump up the bladder). The parameters were adjusted for each patient (Table 2).

A functional respiratory assessment (tidal volume measurement, peak expiratory flow, and oxygen saturation) was performed during spontaneous breathing and using the IAPV. Inspiratory volume, expiratory volume, and peak expiratory flow were evaluated. Tidal volume was assessed in the inspiratory phase (the diaphragm's prevalent muscular activity) and the expiratory phase (elastic return of the lung and chest wall compliance). A day hospital training session was carried out before use at home.

## 3. Results

**Figure 2:**
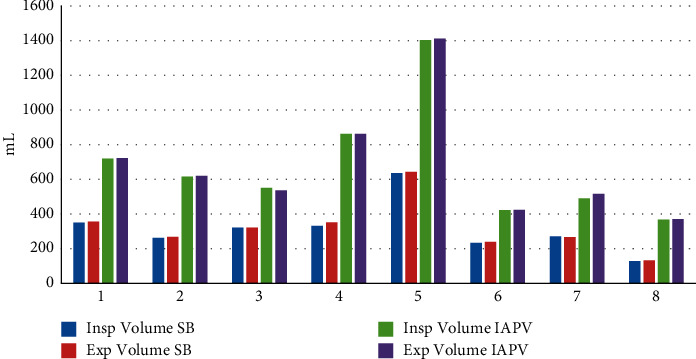
Inspiratory and expiratory volume measurement at baseline and during IAPV.

**Figure 3:**
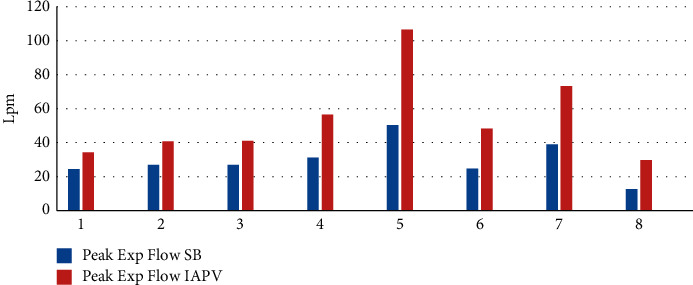
Peak flow at baseline and during IAPV.

All patients performed the baseline assessment and tolerated the IAPV treatment. Pt 1 (congenital myopathy), Pt 2 and 3 (Duchenne patients), and two ALS patients (Pt 4 and 5) had previously refused NIV, while they tolerated and adapted well to IAPV. Pt 6 presented with deep nasal, frontal, and retronucal pressure sores, which interfered with the use of NIV; he therefore enthusiastically accepted IAPV. Pt 2, 7, and 8 presented with aerophagia and gastric overdistension. IAPV, thanks to abdominal compressions, allowed us to counteract the air retention that occurred during noninvasive positive pressure ventilation that Pt 7 and 8 used at night. The mean spontaneous tidal volume at baseline was 316.375 ± 146.80 mL, increased to 678 ± 334 mL using the IAPV. The tidal volume was doubled for all patients during IAPV use. The parameters for each patient are shown in Table 1. Peak expiratory flow measured in baseline conditions was 29.5 ± 10.9 mL. During IAPV, the average peak flow was 54 ± 18.04 mL. Pt 4, 5, 6, 7, and 8 performed air staking during IAPV use. All patients are still using IAPV after three years. Three patients (Pt 1, 2, and 3) rely on the IAPV as their sole method of ventilatory support 24 hours a day. The IAPV, as the only respiratory support, became ineffective for two patients (Pt 4 and 5) after 2 years of use, and these patients then switched to daytime IAPV and nocturnal positive pressure ventilation with a nasal mask due to the appearance of obstructive sleep apnea syndrome. Pt 6, 7, and 8 associated IPAV with nocturnal noninvasive mechanical ventilation with a nasal (Pt 8) or facial (Pt 6 and 7) mask, which they already used (Figures 2 and 3).

## 4. Discussion

We know that the lungs dilate, thanks to the expansion of the thoracic cavity that is realized by two mechanisms: the contraction of the internal intercostal muscles, which raise the ribs and widen the chest (rib or thoracic breathing) and the contraction of the diaphragm, which expands downwards (abdominal or diaphragmatic breathing) [10]. When the diaphragm is weak, a manual or mechanical thrust to the abdominal wall can force the diaphragm upward to expel air below the patient's average resting lung volume or functional residual capacity. Tidal volume improves through several mechanisms: it increases the chest wall elastance because the elastic recoil pressure of the chest wall is negative at this lower lung volume; inspiration takes this increased elastic energy and improves tidal volumes. Also, enhanced length-contraction characteristics of the diaphragm can enhance the force of diaphragmatic contraction. In addition to this, gravity augments both. The patient can further increase tidal volumes and add to ventilator-derived intermittent abdominal pressure ventilation through the respiratory muscles' voluntary activity or by glossopharyngeal breathing.

The LunaBelt device is simple to use, and the corset is quick to put on. It helps to carry out a training period for family members, as for all devices, with particular attention to patients who will have to use noninvasive mechanical ventilation with the mask. The IAPV only operates effectively when the subject is in the sitting posture [2, 5] at an angle of 30° or more and is ideal at 75° [11] because the increase in lung volume is generated by gravity. For severely obese patients or patients with severe chest wall deformity, it may be ineffective. However, there have been reports of patients using IAPV even during sleep with excellent comfort and adherence to treatment [2, 9].

Bach, in 1991, described an extensive series of patients using the IAPV for many years. They suggested that IAPV is a safe and helpful technique of long-term daytime ventilatory support for paralytic/restrictive respiratory deficiency subjects [2]. Its use is enhanced in combination with other noninvasive methods of ventilatory support, thus eliminating the need for tracheostomy and improving the use of glossopharyngeal breathing. Several authors have described follow-up as essential because the IAPV can become less effective over time [2, 5]. We found an improvement in the cough peak, which, in some patients, allowed a better clearance of secretions, even during air staking manoeuvres. Bach and Alba stressed that regular follow-up is essential because IAPV can become less effective with time [2].

Our experience agreed with previous suggestions and results described in recent case reports that patients with severe restrictive syndrome adjusted well to and were successfully ventilated by the IAPV, using it for several years. In our population, IAPV has been well tolerated for over two years. The average time of IAPV use was 10–12 hours per day. In some patients, due to the progression of neurodegenerative disease, it was necessary to integrate the treatment with positive pressure ventilation with a mask during the night hours due to obstructive sleep apnea syndrome. IAPV facilitates diaphragmatic motion and may be particularly useful in patients with bilateral diaphragmatic weakness or paralysis and permits plugging of the tracheostomy tube with cuff deflation for several hours during the day, with the prevention of tracheal damage. IAPV permits patients to speak and provides an effective daytime ventilatory pattern; it also allows the maintenance of an excellent peripheral saturation without dyspnea, a significant improvement in salivary secretion management, and a decrease in the need for tracheal aspiration. IAPV can be used in patients who require NIV many hours a day alternatively or alternating NIV with a mask. NIV can be a cause of severe gastric insufflation. Patients with neuromuscular pathology may have altered intestinal smooth muscle, leading to air retention in the stomach and colonic [12]. In particular, dystrophin is expressed in the smooth muscle of the gastrointestinal tract. The disruption of protein expression can lead to functional disturbances of the gastrointestinal tract, including acute gastric dilatation, gastroparesis, and intestinal pseudo-obstruction [13].

Moreover, aerophagia is a significant NIV-related problem that appears in up to half of patients with NIV and may lead to the discontinuation of treatment. Patients with gastric distension may benefit from the device's abdominal compression during the exhalation phase [9, 10]. Regurgitation of food during meals, catching of clothing on straps and Velcro fasteners, redness of bony prominences, and inability to shower or bathe during use have been reported as possible disadvantages [11]. In the past, sacral decubitus has been described in patients that used IAPV constantly [2].

## 5. Conclusion

The use of IAPV is limited to a few centres, likely due to the long time required to adapt and monitor the patient. It is necessary to have different possibilities for noninvasive mechanical ventilation to guarantee the optimal interface for the patient. IAPV is a comfortable alternative to NIV with a mask, and it is significant for patients requiring daytime support and patients with chronic disease to be considered for NIV. Patients with the need for continuous noninvasive ventilation often present pressure ulcers from the mask, aerophagia, and intolerance to the mask due to interference with social life. These complications can lead to the failure of NIV [14, 15]. IAPV maintains good ventilation and oxygenation and reduces complications related to positive pressure ventilation with a mask. It can also be used often in addition to or alternating NIV with a mask [16]. It can be helpful to alternate the interface in patients who need ventilator support 24 h a day and to carry out daily life activities without interference related to the use of masks.

## Figures and Tables

**Table 1:** Patient's characteristics and slow vital capacity (SVC) at baseline and during IAPV.

Disease	Gender	Age	SVC (ml)	Basal RR	NIV adherence	Pbelt, Ti, FR (cm H_2_O, sec, bpm)	IAPV vital capacity (ml)
Myopathy	F	32	340	15.6	Refused	30, 1.8, 13	748
Duchenne	M	22	270	18.9	Refused	60, 1.5, 15	648
Duchenne	M	20	320	22.2	Refused	50, 1.4, 15	578
ALS	M	62	440	15.4	Refused	30, 1.5, 13	962
ALS	M	63	780	16.9	Refused	50, 1.5, 12	1484
ALS	M	68	250	19.8	Poor compliance	60, 1.2, 18	514
ALS	M	25	280	19.8	Poor compliance	60, 1.5, 14	524
Duchenne	M	19	150	26.2	Poor compliance	50, 1.5, 16	475

**Table 2:** IAPV parameters: we suggest starting with Pbelt of 0–70 Hpa (at the beginning: 30–40 Hpa); select desired Ti (during Ti set, PBAir will be deflated, while the patient will be able to inhale); backup rate as desired; rise time usually 1.0 s; expiratory time (abdominal compression) will be linked to the backup rate and inspiratory time set. For example, set inspiratory time: 1.5 sec, Fr: 15 bpm, and derivative expiratory time: 2.5 sec.

Intermittent abdominal pressure ventilator (LunaBelt)		
Mode	Timed	Spontaneous/timed
Pressure belt	0–70 hPa	0–70 hPa
Time inspiratory	0.3–5.0 sec	Na
Time inspiratory minimum	Na	0.3–3.0 sec
Time inspiratory maximum	[(60/Freq) − 0.6 sec]	[(60/Freq − 0.6 sec)]
Time expiratory minimum	Na	0–1.5 sec
Backup frequency	1–60 bpm	1–60 bpm
Frequency maximum	[60/(Tinsp + 0.6 sec)]	[60/(Tinsp + 0.6 sec)
Rise time	0.1–1.0 sec	0.1–1.0 sec
Trigger inspiratory (nasal cannula)	Na	Auto
Trigger expiratory (nasal cannula)	Na	Auto

## Data Availability

The data that support the findings of this study are available from the corresponding author upon reasonable request.
